# Predicting single-cell gene expression profiles of imaging flow cytometry data with machine learning

**DOI:** 10.1093/nar/gkaa926

**Published:** 2020-10-29

**Authors:** Nikolaos-Kosmas Chlis, Lisa Rausch, Thomas Brocker, Jan Kranich, Fabian J Theis

**Affiliations:** Institute of Computational Biology, Helmholtz Zentrum München, Neuherberg 85764, Germany; Roche Pharma Research and Early Development, Large Molecule Research, Roche Innovation Center Munich, Penzberg 82377, Germany; Institute for Immunology, Medical Faculty, Ludwig Maximilian University of Munich, 82152 Planegg-Martinsried, Germany; Institute for Immunology, Medical Faculty, Ludwig Maximilian University of Munich, 82152 Planegg-Martinsried, Germany; Institute for Immunology, Medical Faculty, Ludwig Maximilian University of Munich, 82152 Planegg-Martinsried, Germany; Institute of Computational Biology, Helmholtz Zentrum München, Neuherberg 85764, Germany; Department of Mathematics, Technical University of Munich, Garching 85748, Germany

## Abstract

High-content imaging and single-cell genomics are two of the most prominent high-throughput technologies for studying cellular properties and functions at scale. Recent studies have demonstrated that information in large imaging datasets can be used to estimate gene mutations and to predict the cell-cycle state and the cellular decision making directly from cellular morphology. Thus, high-throughput imaging methodologies, such as imaging flow cytometry can potentially aim beyond simple sorting of cell-populations. We introduce IFC-seq, a machine learning methodology for predicting the expression profile of every cell in an imaging flow cytometry experiment. Since it is to-date unfeasible to observe single-cell gene expression and morphology in flow, we integrate uncoupled imaging data with an independent transcriptomics dataset by leveraging common surface markers. We demonstrate that IFC-seq successfully models gene expression of a moderate number of key gene-markers for two independent imaging flow cytometry datasets: (i) human blood mononuclear cells and (ii) mouse myeloid progenitor cells. In the case of mouse myeloid progenitor cells IFC-seq can predict gene expression directly from brightfield images in a label-free manner, using a convolutional neural network. The proposed method promises to add gene expression information to existing and new imaging flow cytometry datasets, at no additional cost.

## INTRODUCTION

Extracting actionable knowledge from vast volumes of data acquired with modern high-throughput single-cell profiling methods is an intriguing challenge in the field of computational biology, more so if multiple such methods are to be integrated for one particular biological question. One of the most prominent single-cell profiling methods is fluorescence microscopy (1), which allows for the acquisition of information-rich imaging data. Imaging flow cytometry (IFC) (2) is a key extension of fluorescence microscopy that combines the high-throughput capabilities of flow-cytometry (3) with imaging at the single-cell level. IFC datasets have three main characteristics that make them well-suited for quantitative analysis. First, fluorescent markers can be used to label distinct cellular characteristics and functions, rendering the generated datasets rich in information. Second, each cell is imaged separately. As such, there is no need for a segmentation method in downstream analysis steps at the cost of losing information regarding the original morphology of the tissue. Third, the high-throughput nature of imaging flow cytometry allows for the imaging of a very large number of cells (tens of thousands or more) per experiment in a standardized fashion. High-throughput image acquisition naturally leads to large datasets, which calls for contemporary analysis methods in particular machine learning for analysis and interpretation.

As an extension of flow cytometry, IFC has the potential to tackle diagnostic applications in a clinical setting. Flow cytometry is a key technology used to diagnose and evaluate hematopoietic neoplasia (4). While historically, diagnosis of such malignancies relied strongly on morphological changes of malignant cells, modern diagnostics combines morphological assessment with immunophenotyping and genetic analysis (5). The large heterogeneity of lymphomas and leukemias require a precise characterization of neoplastic cells, hence a large panel of specific antibodies is required for reliable diagnosis (6). Recently, deep learning analysis of histology imaging data has gained attention from clinicians and pathologists in the diagnosis of cancers. Convolutional neural networks have achieved a success rate in the classification of certain tumors that match the success rate of pathologists (7,8). Data obtained by IFC is ideally suited for deep learning-assisted image analysis and hence can be a valuable tool in the diagnosis of lymphomas and other diseases affecting blood cells, such as immunodeficiencies.

IFC allows for imaging of cells and studying cellular properties through corresponding surface markers. As the measurement of surface markers occurs via fluorescently labeled antibodies, this measurement is naturally limited by the number of available fluorescent channels. In turn, this limits the cellular diversity that can be studied using a standard IFC approach. Additionally, the view of the dataset is inherently biased since the surface markers are selected prior to performing the experiment. In contrast, direct observation of each cell's molecular properties would allow for an unbiased view of each cell's inner workings. A natural example of such a high-throughput unbiased view of cellular properties is single-cell omics (9). Specifically, single-cell transcriptomics (SCT) (10,11) corresponds to an additional modality of information-rich and high-throughput datasets at the single-cell level. The novelty of SCT methods lies in their ability to measure the full gene expression profile of each individual cell. As a result, the advent of single-cell transcriptomics has led to new advancements in several areas of biology, such as hematopoiesis (12,13), embryogenesis (14,15), the airway epithelium (16,17) and the immune system (18–20). With increasing complexity and size of these data sets (10), these biological advancements have gone hand-in-hand with the development of novel statistical and machine learning concepts for analyzing SCT data (21–24).

Machine learning approaches have also been developed for the analysis of IFC measurements, mainly focussing on the identification and automated sorting of different cell types (25–28). Nonetheless, recent developments in machine learning methods have shown that analysis of imaging data can be extended far beyond simple sorting of cell types. For example, it was recently demonstrated that gene mutational status can be predicted from imaging data (7). Moreover imaging data can be used in order to estimate the cell-cycle stage (27) and to predict cellular decisions such as differentiation (29) based on morphology information. Additionally, technologies that offer both IFC and SCT capabilities are expected in the near future (30,31). Datasets including both imaging and transcriptomic views of each individual cell will offer unprecedented quality and quantity of information. As such, they can aid our understanding of biological systems. Having SCT information available in IFC experiments would not only alleviate the bias of preselecting surface markers prior to performing the IFC experiment, but would additionally allow studying cellular properties and functions in unprecedented resolution by providing expression information for individual cells.

However, at the moment the IFC and SCT modalities are still acquired separately, in different experiments and for different populations of cells. In this paper we introduce IFC-seq: a machine learning methodology for predicting the expression profile of each individual cell of an IFC experiment, based on integrating a corresponding SCT experiment that includes the same cell types of interest. We demonstrate that our method correctly predicts and localizes the expression of key marker genes for each cell type. We also demonstrate that in some cases, the estimation of gene expression can be performed in a label-free manner from the brightfield images only, based on the morphology of each cell (32). To the best of our knowledge, this is the first study that aims to computationally augment IFC datasets with an additional SCT information modality.

**Figure 1.**
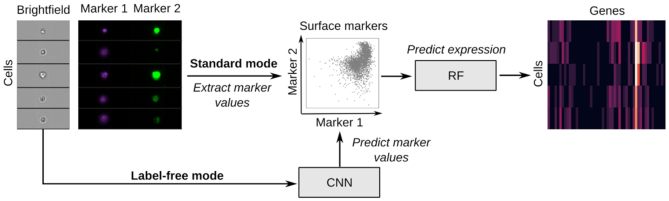
IFC-seq predicts gene expression of individual cells in IFC datasets. Given the values of the surface markers of the IFC experiment, IFC-seq proceeds to predict the gene expression profile of each cell. Expression prediction is achieved via a Random Forest (RF) for regression. In standard mode, the measured values of the markers are utilized, while in label-free mode the markers are predicted from the cells’ morphology using the brightfield images and a convolutional neural network (CNN). Learning the correspondence between marker values and expression becomes possible by co-registering an independent single-cell transcriptomics experiment where the same surface markers are also measured for each cell.

The closest method to our approach is (7) where the imaging modality is used to predict the mutation status of select genes. Nonetheless, IFC-seq differs from (7) in the following points: First, unlike the case of (7) where ground truth mutational status is available for each sample, in the case of IFC-seq no ground truth expression information is available for the IFC experiments. Second, IFC-seq predicts continuous expression instead of a binary outcome. Third, IFC-seq predicts the expression of hundreds of genes that correspond to population markers, instead of predicting a small number of predetermined genes. The workflow of IFC-seq is demonstrated in Figure 1.

## MATERIALS AND METHODS

### Preprocessing the SCT datasets

Each SCT dataset was pre-processed before being co-registered to its corresponding IFC dataset. First, the surface markers values of the SCT datasets were normalized to [0,1]. Then, genes that were expressed in fewer than 20 cells of the SCT experiment were excluded from further analysis. Next, the expression of all genes was logarithmized using the natural logarithm. Subsequently, gating was performed by an expert on the surface marker values of the SCT dataset in order to identify the cellular subpopulations of interest. It should be noted that gating information is not used in the predictive model, but is only used to validate the model's results. Last, a set of top 100 differentially expressed marker genes was computed for each cellular subpopulation, using the ‘rank_genes_groups’ function of Scanpy (24). As such, differentially expressed genes were identified in a purely unbiased data-driven manner and no manual identification of marker genes was performed by an expert.

### SCT—human cord blood mononuclear cells

The first SCT dataset corresponds to Cord Blood Mononuclear Cells (CBMCs) (33). The original dataset includes human and mouse cells and the count matrix and surface markers are available as supplementary files GSE100866_CBMC_8K_13AB_10X-RNA_umi.csv.gz and GSE100866_CBMC_8K_13AB_10X-ADT_umi.csv.gz, respectively. Only human cells were kept by selecting cells that express more human than mouse genes. To be precise, human genes in the count matrix are characterized by a ‘HUMAN_’, while mouse genes are characterized by a ‘MOUSE_’ prefix in the gene name. We identified as human cells, the cells that express more human than mouse genes. We considered a gene to be expressed in a cell if it corresponds to non-zero counts. Additionally, cells expressing fewer than 200 genes were discarded from further analysis and highly variable genes were kept using the filter genes dispersion method of Scanpy (24) with parameters min mean = 0.0125, max mean = 3, min disp = −0.15. All subsequent preprocessing steps were performed as described in the previous section. After preprocessing, the dataset includes 8017 human cells and 2768 genes, as well as CD3 and CD8 surface marker values measured for each cell. Subsequently, the cells were sorted into helper T-cell (CD3+CD8-) and cytotoxic T-cell (CD3+CD8+) sub-populations based on the CD3 and CD8 marker values.

### SCT—mouse myeloid progenitor cells

The second SCT dataset corresponds to a publicly available dataset of mouse myeloid progenitor cells (13). After preprocessing it includes 2730 cells with 3371 genes, as well as FcgR and CD34 surface marker values for each cell. The preprocessing steps were performed as described above. Gating was subsequently performed to sort the cells into three sub-populations: Common Myeloid Progenitor (CMP) cells, Granulocyte/Macrophage Progenitor (GMP) cells and Megakaryocyte/Erythrocyte Progenitor (MEP), using the same gates as in (13).

### IFC—mice

Sex and aged matched (8 weeks) C57BL/6 mice were purchased from Envigo. The permissions for animal experiments were granted by the animal ethics committee of the Regierung von Oberbayern, Munich, Germany.

### IFC—human peripheral blood mononuclear cells

IFC was used to acquire data of human Peripheral Blood Mononuclear Cells (PBMCs). The resulting IFC dataset corresponds to 82 109 human PBMCs with CD3 and CD8 surface marker measurements for each cell. Gating on the CD3 and CD8 markers was employed to sort the cells into helper T-cell (CD3+CD8-) and cytotoxic T-cell (CD3+CD8+) sub-populations.

Data acquisition was performed as follows: Blood from healthy donors was diluted in Phosphate-Buffered Saline (PBS) carefully layered onto a Ficoll cushion (Biocoll: Density 1.077 g/ml). After centrifugation the layer containing PMBCs was collected and washed. 5 × 106 cells were stained with CD3 PE-Cy7 (clone UCHT1, Biolegend), CD8a-AF647 (clone RPA-Ta, Biolegend) and live dead fixable violet dye (ThermoFischer). After fixation (4% PFA, 10 min) cells were analyzed by imaging flow cytometry. After acquisition, TIF-images (32 × 32 pixels, 16-bit, raw) of live dead-CD3+CD8a−, live dead-CD3+CD8a+ and live dead-CD3−CD8a− were exported and used for analysis and the CD3 and CD8 surface markers were normalized in [0,1].

### IFC—mouse myeloid progenitor cells

Two separate IFC datasets were acquired for this study. The training dataset was used to train a CNN for label-free marker prediction consists of 65 008 cells. The test set was used to evaluate the results of IFC-seq and consists of 3137 cells. Both IFC datasets include brightfield, FcgR and CD34 images of cells, along with the measured CD34 and FcgR surface marker intensity values. Subsequently, CMP, GMP and MEP cells were identified by gating on the CD34 and FcgR markers.

The data acquisition process was the following: BM cells were flushed from femur and tibia with PBS + 2% fetal calf serum (FCS) using syringes. Erythrocytes were lysed using an ammonium chloride potassium buffer. Number of live cells was determined using a CASY cell counter (OMNI Life Science). 5 × 10^6^ cells were stained with CD117 APC (clone 2B8, eBioscience), CD34 FITC (clone RAM34, eBioscience), Sca-1 PE-Cy5 (clone D7, eBioscience), FcgR PE-Cy7 (clone 93, Invitrogen) and Lin-1 BV421 (Biolegend) and analyzed on an ImageStreamX MKII imaging flow cytometer (Luminex). TIF-images (32 × 32 pixels, 16-bit, raw) of Lin-1-CD117+Sca-1+FcgR-CD34− MEP, Lin-1-CD117+Sca-1+FcgRintCD34int CMP, Lin-1-CD117+Sca-1+FcgR+CD34+ GMP cells were exported and used for analysis and the CD34 and FcgR surface markers were normalized in [0,1].

### Predicting gene expression

The goal of the proposed IFC-seq method is to augment IFC datasets with expression information at the single-cell level. Ideally, that would require data where the imaging modality and gene expression are available for the exact same cell. However, while such promising techniques have been proposed (30,31) they are not yet well established and broadly available. Thus, IFC-seq overcomes the lack of such datasets by co-registering an IFC experiment to a corresponding SCT experiment that includes a common subset of cell-types. The co-registration step is made possible by aligning the datasets using surface markers that are present in both the IFC and SCT modalities. That is, we assume that if a cell in the IFC experiment is close in the space of surface markers to cells in the SCT dataset, then its expression can be estimated from the expression of its corresponding cells in the SCT dataset. To ensure that surface marker values are comparable across modalities, as part of co-registration we independently normalize each marker within each modality so that its range of values extends from 0 to 1.

Consequently, we treat expression prediction as a regression problem and predict thousands of genes per cell, given the values of the corresponding surface markers. Specifically, the scikit-learn (34) implementation of a Random Forest for regression (35) was employed. The Random Forest was configured to minimize the mean absolute error, ‘max_features’ was set to ‘sqrt’ and the ensemble consisted of 50 trees. The Random Forest was trained separately for the human and mouse test cases. In the case of mouse data the CD34 and FcgR markers are used as input while CD3 and CD8 were used for the human data. Additionally, surface marker CD4 is directly predicted for the human data along with gene expression, since surface CD4 is a known helper T-cell marker but the correlation between the measured protein and transcript CD4 levels is low (33). Each SCT experiment is split into a 70% training set and a 30% test set. No validation set was used when training the Random Forest, since no hyperparameter tuning was performed. The trained Random Forest model is trained on the SCT dataset, it is then employed to predict the expression of the corresponding (human or mouse) IFC dataset.

### Predicting surface markers in a label-free manner

**Figure 2.**
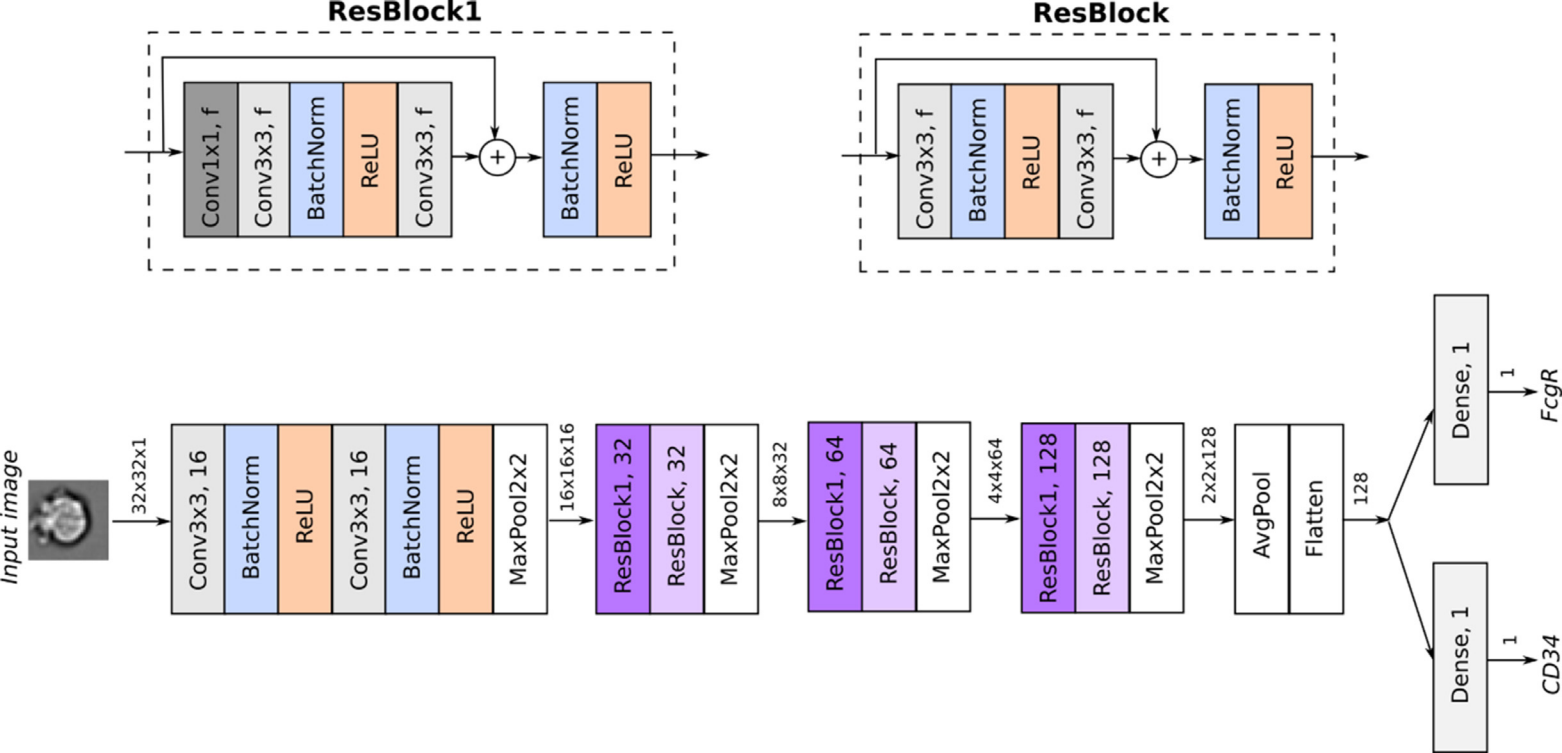
Overview of the architecture of the CNN for regression used to predict the surface markers in the mouse data. Given the brightfield image of each cell, the network predicts the value of the CD34 and FcgR surface markers, whose values are continuous and lie in [0,1]. After the initial input stem, the network employs residual blocks whose architecture is visualized on the top part of the figure. Every convolutional layer employs zero padding. Thus, the spatial dimensions of the intermediate tensors are only reduced via pooling operations. Tensors are visualized as arrows, along with their dimensions (height × width × channels).

In the label-free mode of IFC-seq, a Convolutional Neural Network (CNN) (36) was employed to predict the surface marker values based only on the 32 × 32 brightfield image of each cell in the IFC experiment. Since network architectures that perform well on natural images have been shown to perform well on IFC data (27), we based our approach on the popular residual CNN architecture which achieves state of the art results on natural images (36). It should be noted that label-free prediction is only expected to work if there is sufficient morphological information in the brightfield images of the cells. As such, we will demonstrate the label-free mode IFC-seq in the case of the mouse dataset, since there is no sufficient morphological difference between the helper and cytotoxic T-cells of the human dataset. The CNN was trained using Adam (37) for 50 epochs using a batch size of 64 on the IFC training dataset of 65 008 mouse myeloid progenitor cells, while 10% of the IFC dataset was randomly left out of training and was used for validation. The best model according to the validation loss was saved. Additionally, early stopping with a patience parameter of five epochs was employed during training. Moreover, data augmentation was employed on the training set. Such augmentation corresponds to flipping the images along the vertical, horizontal, or both axes. The network consists of 17 convolutional layers and ∼700 000 parameters. Each activation layer, except the last, is preceded by a batch normalization layer (38). The neural network was implemented in Keras. An overview of the CNN architecture is presented in Figure 2 and the trained model is available online at https://github.com/theislab/ifcseq.

## RESULTS

### Overview

Next, we will proceed to demonstrate the results of IFC-seq on the human and mouse test cases. For each of these two test cases, the process is the following: First, we will evaluate the predicted expression on the left out test set of the SCT dataset. This is helpful since we have ground truth expression that we can compare to. Thus, this will allow for the quantification of the model's predictive capability and provide an upper bound for its expected performance when applied to the IFC data. In both cases, we will demonstrate that while IFC-seq can be used to predict all genes that are included in the SCT experiment, prediction performance is not uniform across all genes. Specifically, gene expression is only predicted successfully for marker genes of the cellular subpopulations of interest.

Furthermore, we will apply IFC-seq and predict expression for the corresponding IFC seq experiment. Since no ground truth expression is available for each cell in the IFC data, we need to employ a different validation approach. That is, we will assess the predicted expression at the population level and quantify to what extent the predicted expression of population-specific marker genes follows the same pattern as observed in the SCT experiment. That is, if IFC-seq is successful, then the expression patterns of population-specific marker genes should be consistent across the IFC and SCT modalities. As mentioned in the previous section, we will also demonstrate the label-free capability of IFC-seq and predict gene expression directly from the brightfield images in the case of mouse cells. On the other hand, IFC-seq label-free mode is not supported in the case of human blood cells, since the T-cell subpopulations of interest cannot be distinguished by morphological features alone.

### Human blood mononuclear cells

IFC-seq was employed to predict gene expression of human blood mononuclear cells, based on the measured CD3 and CD8 markers for each of the SCT and IFC modalities. It should be noted that the SCT dataset consists of CBMCs while the IFC dataset consists of PBMCs. However, they both include helper and cytotoxic T-cell subpopulations. Additionally, the bulk of ‘other’ cells is considered as a subpopulation where T-cell specific markers are not expected to be expressed. As such, the results of IFC-Seq are assessed with respect to these three cellular subpopulations.

**Figure 3.**
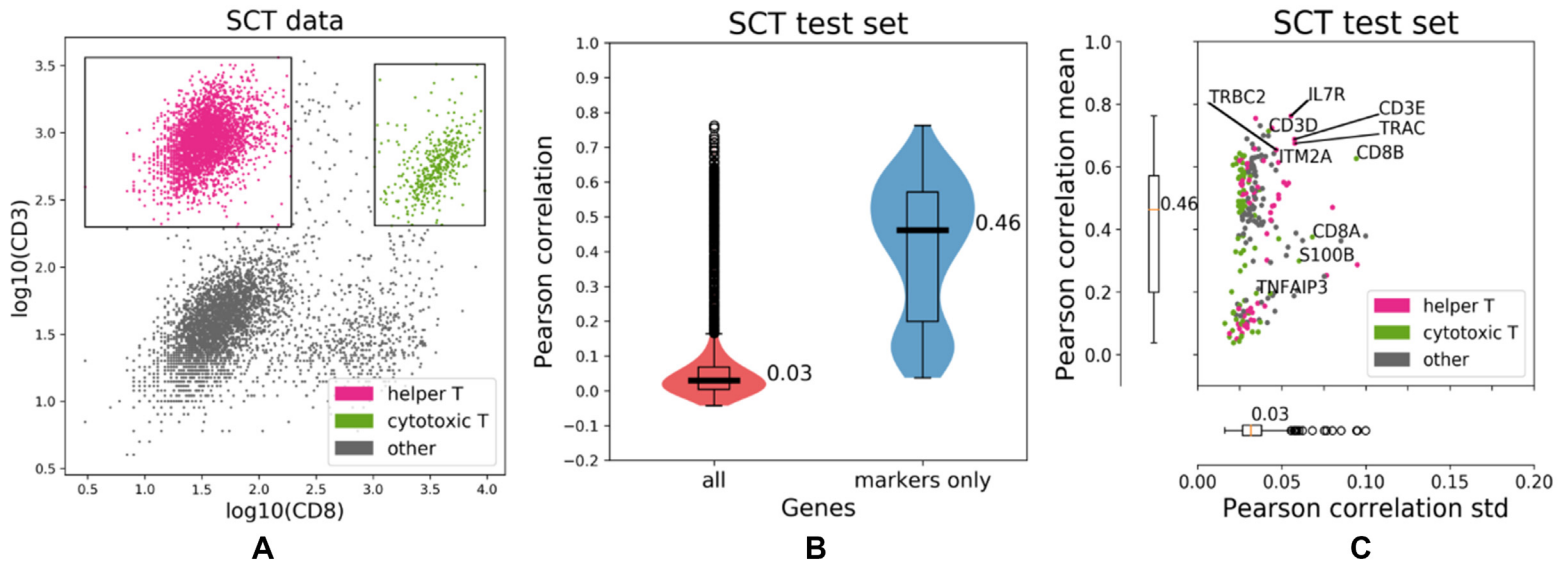
IFC-seq results on SCT data of human blood mononuclear cells. (**A**) The SCT dataset and corresponding helper and cytotoxic T-cell gates plotted on top of the CD3 and CD8 markers. The population of ‘other’ cells corresponds to all unknown cell-types that are not included in the population-specific gates. (**B**) The predicted gene expression is more accurate for the differentially expressed population-specific marker genes than for all genes present in the SCT dataset, as quantified by the Pearson correlation between the true and predicted expression values for each cell in the SCT test set. (**C**) Looking at the population-specific marker genes shows that they are predicted with low uncertainty, as quantified by the standard deviation of the per-gene Pearson correlation computed over the trees in the Random Forest ensemble. Individual points correspond to distinct population-specific marker genes. Genes are colored according to the respective population they are markers for. Select population markers are overlaid on top of the scatter plot.

As seen in Figure 3, IFC-seq successfully predicts gene expression in the case of marker genes for the subpopulations of helper and cytotoxic T-cell. These marker genes correspond to the differentially expressed genes identified during preprocessing (see Materials and Methods). Overall, the median Pearson correlation between the predicted and true expression is 0.46 when considering only the top 100 genes per subpopulation, while it drops to 0.03 if all genes in the SCT dataset are taken into account. Moreover, the top marker genes are predicted with low uncertainty, as quantified by the standard deviation of the Pearson correlation achieved by individual trees in the Random Forest ensemble, which equals to 0.03. Table 1 summarizes the median Pearson and Spearman correlations, as well as the mean squared error achieved by the Random Forest, as well as a linear regression baseline model. Last, the correlation between the CD3 and CD8 surface markers and their corresponding coding genes is visualized in Supplementary Figure S1 in the supplement.

**Table 1.** Predictive performance of Random Forest regression and Linear Regression on the SCT test of human blood mononuclear cells. The median value of each statistic across all cells is reported

Method	Pearson correlation	Spearman correlation	Root mean squared error
Random Forest	0.46	0.46	0.52
Linear regression	0.38	0.35	0.52

**Figure 4.**
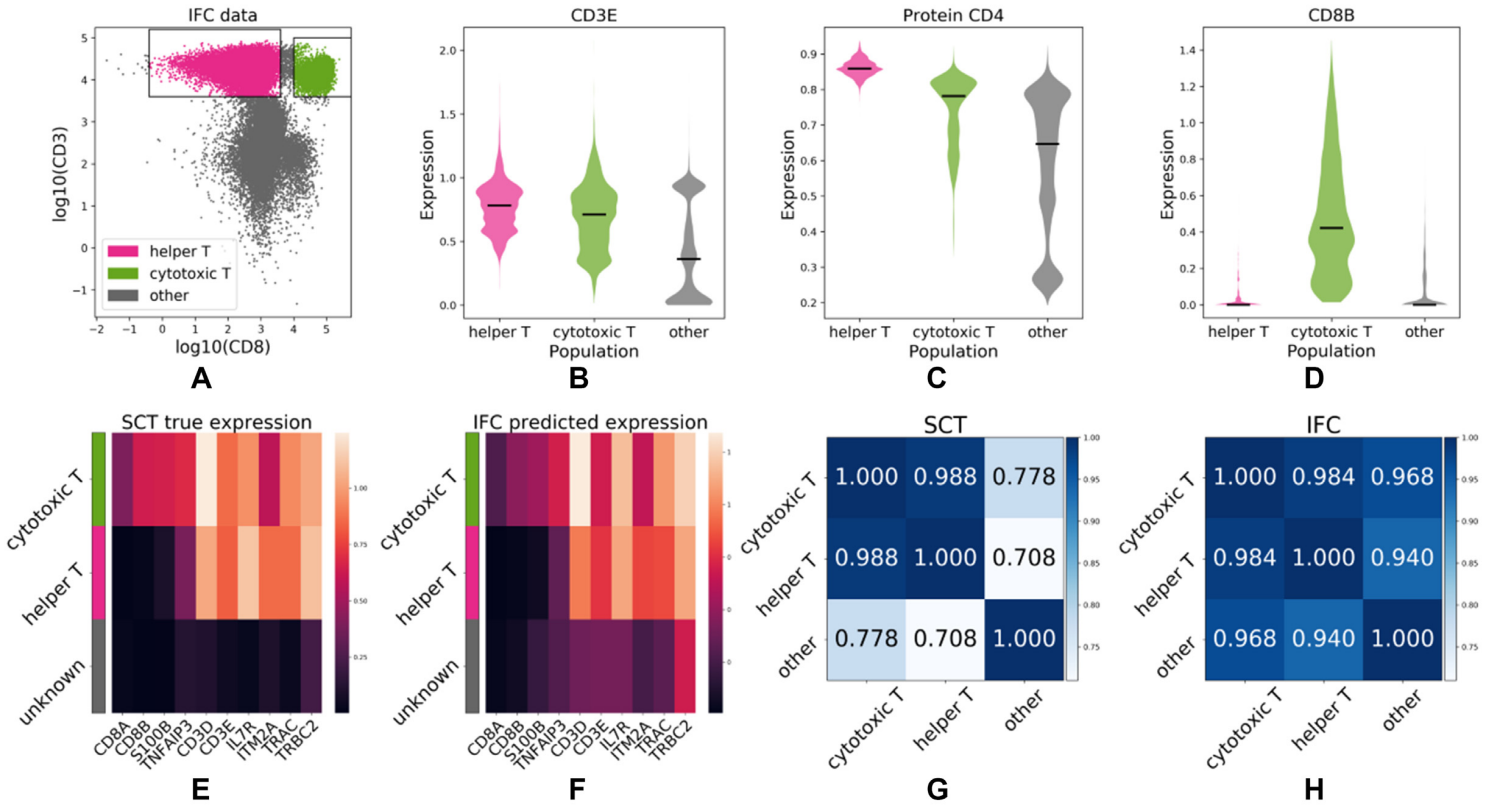
IFC-seq predicts gene signatures of helper and cytotoxic T-cell subpopulations in human blood mononuclear cells. (**A**) IFC dataset and corresponding gates for helper and cytotoxic T-cells on the CD3 and CD8 markers. The population of ‘other’ cells corresponds to all unknown cell-types that are not included in the population-specific gates. (**B**) CD3E, a known T-cell marker is predicted to be highly expressed in both helper and cytotoxic T-cells. (**C**) Surface CD4, a helper T-cell marker, is predicted to be predominantly expressed in helper T-cells. (**D**) CD8B, a cytotoxic T-cell marker, is predicted to be almost exclusively expressed on cytotoxic T-cells. (**E, F**) The expression profiles between marker genes agree across the SCT and IFC experiments. Each row of the heatmap corresponds to a population while each column corresponds to the expression of a gene averaged across all cells in a population. (**G, H**) The transcriptional similarity of populations is similar across the SCT and IFC modalities. That is, cytotoxic and helper T-cells are more similar to each other than the bulk of other cells.

When applied to the IFC dataset, IFC-seq correctly predicts that the CD3D and CD3E are highly expressed in the helper and cytotoxic T-cells. This is to be expected since both CD3D and CD3E correspond to proteins necessary for T-Cell receptor signalling (39). Moreover, surface CD4, a known helper T-cell marker, is predicted to be highly expressed in the helper T-cells. It should be noted that CD4 was not included in the set of markers measured experimentally for the IFC experiment, so this successful prediction of IFC-seq is exclusively data-driven. Nonetheless, while CD4 is predicted to be less expressed in the cytotoxic and other cells, it should ideally be predicted to be closer to zero for these subpopulations. Moreover, CD8A and CD8B are two known cytotoxic T-cell markers (40) that IFC-Seq predicts to be highly expressed almost exclusively in this subpopulation. The above results are visualized in Figure 4(A-D).

Additionally, IFC-Seq correctly predicts the expression patterns of several other genes that are known to be associated with the subpopulations of interest. These marker genes include: S100B which is associated with T-cells and natural killer cells (41), TNFAIP3 which is related to immune response (42), CD27 which associated with T-cell immunity (43), ITM2A which is involved in T-cell activation (44), IL7R which is known to be expressed in naive T-cells (45), TRBC2 and TRAC which are related to the T-cell receptor (46), CD69 and SELL, associated with both helper and cytotoxic T cells (47) and finally SOX4 which is associated with helper T-cells (48).

Figure 4E and F visualizes the expression profiles of the aforementioned markers across the SCT and IFC modalities. While gene expression is predicted at the single-cell level, the figure visualizes the average expression per population in order to highlight patterns at the population level. By observing these patterns it is straightforward to distinguish the helper and cytotoxic T-cells from each other, as well as from the bulk of other cells. To be precise, CD8A, CD8B, S100B and TNFAIP3 only mark cytotoxic T-cells, while the remaining markers separate helper T-Cells from the bulk of other cells. Additionally, the transcriptional similarity of populations is quantified as the Pearson correlation of the population-average expression of all top marker genes across the populations. That is, the helper and cytotoxic T-cells are expected to be transcriptionally more similar to each other, than to the bulk of other cells. That is indeed the case when the similarity is predicted with true expression in the SCT experiment and with IFC-seq predicted expression for the IFC data, as shown in Figure 4G and H. However, in the case of predicted expression for the IFC data the differences in population similarities are not as pronounced. Nonetheless, by calculating the 95% confidence intervals for the Pearson correlations via Fisher's transformation (49), we see that the similarity of cytotoxic and helper T-cells is at least 0.979 (low confidence interval). On the other hand, the similarity of cytotoxic T-cells to other cells is at most 0.975 and the similarity of helper T-cells to other cells is at most 0.953 (high confidence intervals). As such, cytotoxic and helper T-cells are significantly more similar to each other than to the bulk of other cells, even when looking at the predicted expression profiles of the IFC data.

### Mouse myeloid progenitor cells

Next, we present the results of IFC-seq on mouse myeloid progenitor cells where gene expression was predicted based on the CD3 and CD8 markers for each of the SCT and IFC modalities. In the case of IFC, we present the results when the measured marker values are employed, as well as the case where IFC-seq is performed in label-free mode and the markers are predicted from directly from the brightfield images.

**Figure 5.**
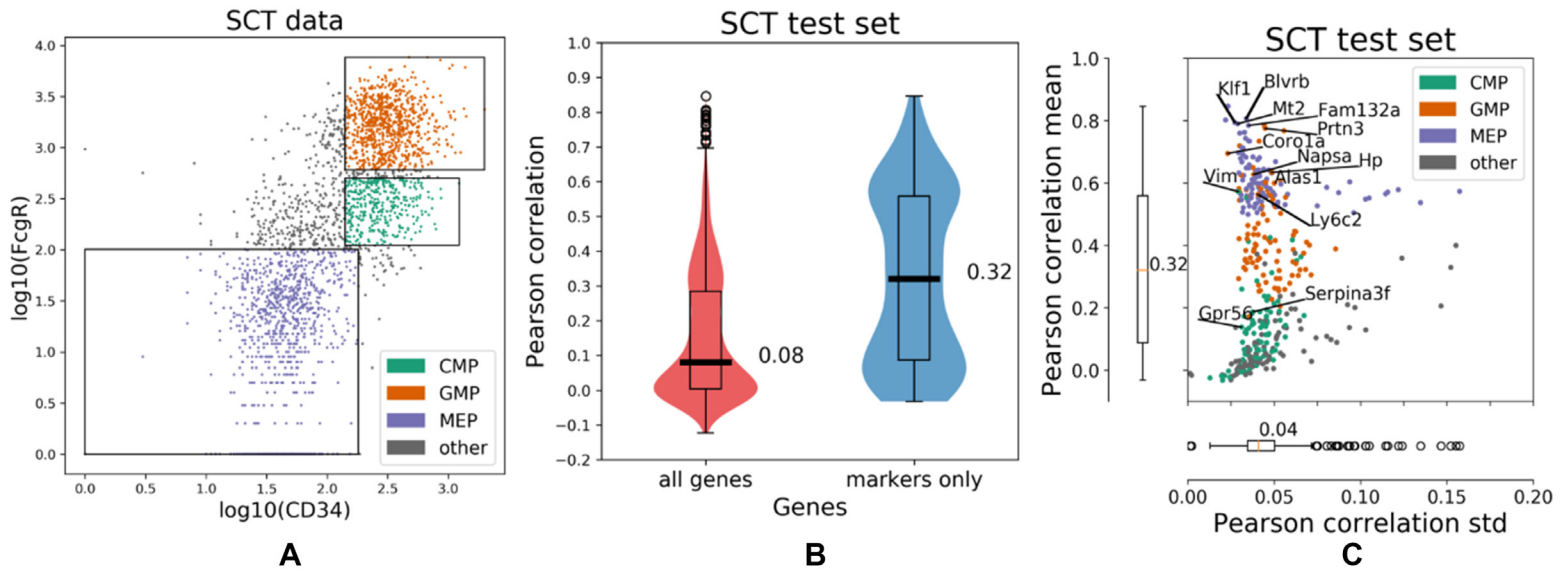
IFC-seq results on SCT data of mouse myeloid progenitor cells. (**A**) SCT data plotted on top of the CD34 and FcgR markers, along with gates for the CMP, GMP and MEP populations of interest. The population of ‘other’ cells corresponds to all unknown cell-types that are not included in the population-specific gates. (**B**) The gene expression is predicted more accurately for the population-specific marker genes, compared to the bulk of all genes in the SCT experiment. Specifically, the median Pearson correlation between the true and predicted expression iis 0.32 for the marker while it drops to 0.08 when all genes are considered. (**C**) The population-specific marker genes are predicted with low uncertainty of 0.04, as quantified by the standard deviation of the Pearson correlation achieved by individual trees in the Random Forest ensemble. Individual points correspond to distinct population-specific marker genes. Genes are colored according to the respective population they are markers for. Select population markers are overlaid on top of the scatter plot.

**Figure 6.**
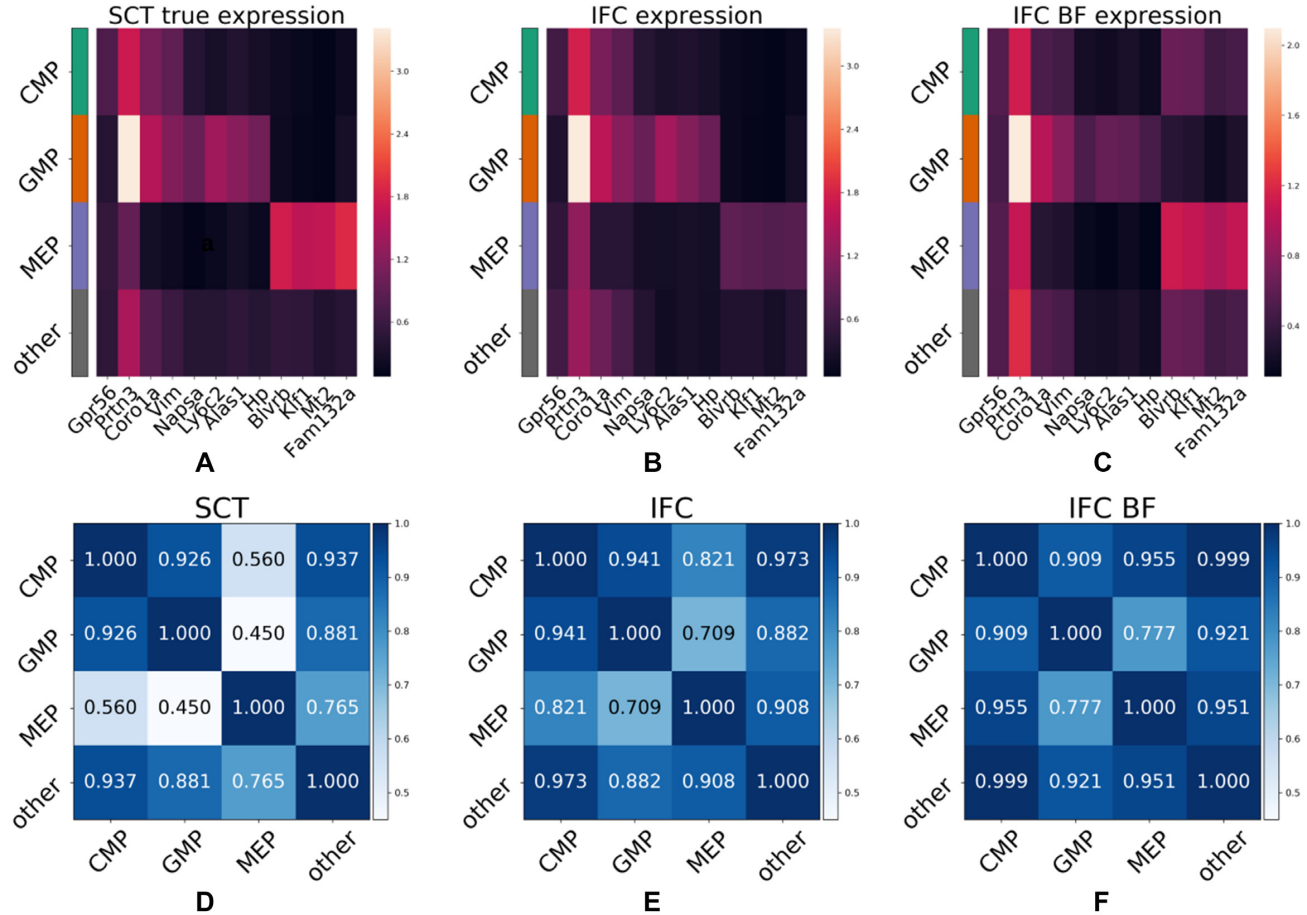
**I**FC-seq predicts gene signatures of mouse myeloid progenitor cells. (A–C) Heatmaps of average gene expression for population-specific marker genes. The true and predicted expression profiles for (**A**) SCT and (**B**) IFC, respectively, are in agreement. (**C**) In the case where IFC-seq is performed in label-free mode, it is still possible to distinguish CMP, GMP and MEP cells from each-other, but it is harder to separate CMP cells from the background of all other cells in the experiment. (D–F) The transcriptional similarity of populations shows the same picture as the previous heatmaps, in a quantified manner. (**D**) CMP cells are transcriptionally similar to the background population of other cells even when considering the true expression of the SCT experiment. Additionally, CMP cells are more transcriptionally similar to GMP than to MEP cells. A similar pattern is visible in the case of predicted expression for the IFC data using the true marker values (**E**) and is still noticeable although noisier in the case of label-free mode (**F**).

Similar to the case of human cells, the predicted gene expression is more closely correlated to true expression when focusing only on population-specific marker genes, instead of all genes in the SCT dataset. That is, when looking only at markers the median Pearson correlation between true and predicted expression is 0.32, as opposed to 0.08 when looking at all genes. Additionally, the population-specific marker genes are predicted with low uncertainty, as the median standard deviation of the per-gene Pearson correlation is only 0.03. The aforementioned results are presented in Figure 5, while the relationship between the CD34 and FcgR markers and their respective coding genes is presented in Supplementary Figure S2 of the supplement. Interestingly, the prediction quality of the model appears to be population-specific, contrary to what was observed for the human data. However, unlike the human data where helper and cytotoxic T-cells correspond to distinct clusters in the space of the surface markers, the subpopulations of the mouse data correspond to a continuous differentiation process. Gene expression is best predicted for the MEP marker genes, which agrees with the observation that MEP cells yield more distinct expression profiles (Figure 6D). On the other hand, gene expression is not predicted as well for the CMP marker genes. This could be explained by the fact that the CMP cells lie in a smaller range of the surface markers than the GMP and MEP cells, which could result in reduced sensitivity of the model in that area of the feature space. Last, the median Pearson correlation, Spearman correlation and the mean squared error achieved by the Random Forest, as well as a linear regression baseline model are presented in Table 2.

**Table 2.** Predictive performance of Random Forest regression and linear regression on the SCT test of mouse myeloid progenitor cells. The median value of each statistic across all cells is reported

Method	Pearson correlation	Spearman correlation	Root mean squared error
Random Forest	0.32	0.35	0.40
Linear regression	0.32	0.35	0.39

We subsequently apply IFC-seq on the corresponding IFC dataset two times, in standard and in label-free mode and compare the results. In label-free mode, the CD34 and FcgR markers are predicted with a CNN (see Materials and Methods). The performance of the CNN on the IFC dataset, corresponding to the Pearson correlation between the true and predicted marker values, is 0.38 ± 0.16 for CD34 and 0.5 ± 0.016 for FcgR. The standard deviation was calculated using 10 000 bootstrap iterations (50).

Examining the results on the mouse data shows that IFC-seq successfully predicts the expression of key marker genes for the subpopulations of interest in the IFC dataset, purely in a data-driven manner. Specifically, IFC-seq is successful at predicting the expression of known CMP markers, such as Serpina3f (51,52) and Gpr56 (53). Next, IFC-seq predicts the expression of GMP markers, such as Napsa, Ly6c2, Alas1, Hp, as well as known GMP markers Coro1a (54), Ly6c2 (55), Vim (56) and Prtn3 (55). Additionally, some GMP markers like Coro1a and Vim are also expressed in the progenitor populations of CMP cells. Next, MEP markers predicted by IFC-seq include Mt2, Fam132a and known MEP markers Blvrb (54) and Klf1 (57).

Figure 6A–C visualizes gene expression averaged per population, while the gating strategy for the IFC data is shown in Supplementary Figure S3. Visual inspection of the gene expression heatmaps highlights agreement between the population specific gene expression patterns across the modalities of true SCT expression and predicted expression for the IFC experiment (both in standard and label-free modes of IFC-seq). That is, it is straightforward to separate the CMP progenitor cells from their descendant populations of GMP and MEP cells, as well as GMP and MEP cells from each other, based on the population-specific markers mentioned above. As expected, there is loss of information when IFC-seq is performed in label-free mode. Nonetheless, it is still possible to easily distinguish GMP and MEP cells based on expression predicted from morphological information alone. It is also possible to distinguish CMP cells from their two descendant populations. However, in label-free mode the predicted expression profile of CMP cells is close to the profile of the background population of other cells. Nonetheless, it should be noted that the CMP cells and background population have similar expression profiles even in the case of true expression in the SCT experiment, as seen in Figure 6D.

## DISCUSSION

In this paper, we introduced IFC-seq: a machine learning methodology which can augment IFC datasets by predicting an additional modality of single cell transcriptomics for each cell at no additional cost. Predicting the expression profile of key marker genes for each single cell in the IFC experiment is made possible by coupling it to a corresponding independently acquired SCT experiment using common surface protein markers. Additionally, we showed that for the SCT datasets, where ground truth expression is available, IFC-seq is successful at predicting the expression of population specific marker genes with low uncertainty. Naturally, since the model's predictions are based on surface markers, it performs better for genes associated with populations characterized by these markers. Last, we also showed that in some cases, such as the mouse bone marrow cells where morphology is informative, it is possible to directly predict the gene expression of population-specific marker genes for each cell in a label-free manner. This label-free mode of IFC-seq is made possible by using the brightfield images of the IFC experiment and leveraging a convolutional neural network as an additional step. The main goal of this study is to provide a proof of concept that demonstrates the feasibility of predicting gene expression of key marker genes in IFC data by aligning an independent SCT experiment with overlapping cellular sub-populations. To this extent, we provide the code for IFC-seq and all data used in this publication online at https://github.com/theislab/ifcseq.

In both test cases of human blood, as well as mouse bone marrow cells the proposed IFC-seq methodology successfully predicted key gene markers of the populations of interest. These results are promising considering the underlying limitations, such as the low resolution of the IFC images (32 × 32 pixels) and the complexity introduced by the co-registration step used to couple the independent SCT and IFC experiments via a limited subset of common surface markers. The similar predictive performance of the Random Forest and linear regression within the SCT modality suggests that non-linear effects are not a bottleneck in model accuracy. Such a bottleneck is potentially posed by the fact that gene expression was predicted only from two available markers in each dataset. Since the performance of IFC-seq depends on the selection of surface markers, it is only applicable in cases where markers for the cellular populations of interest are known and available during model training. Additionally, IFC-seq is sensitive to batch effects related to intra-modality variability across independent experiment replicates (58), as well as inter-modality variability of the surface markers across the SCT and IFC modalities. In this study, we alleviated the inter-modality variability by marker normalization. Moreover, we expect that upcoming batch effect correction methods (59,60) will further alleviate challenges related to both intra- and inter-modality variabilities. While performance of IFC-seq is bound by the co-registration step, we expect that if more relevant surface markers become available, the predictive capability of IFC-seq will improve. Moreover, augmenting IFC datasets with information of gene expression at the single-cell level, can substantially increase the depth of available information, supplementing the measured surface protein markers. In fact, cell states are often determined by biological processes that might not be identified by surface markers alone, yet show distinct transcriptional signatures. It is worth noting that augmenting IFC datasets with the proposed method comes at zero additional cost, assuming that the markers coupling the IFC to the corresponding SCT experiment are available or that morphological information is sufficient in order to apply IFC-seq in label-free mode.

Predicting gene expression reduces the need for surface markers in certain use cases and that is useful for two main reasons. First, the number of available fluorescence channels is always limited. By being able to predict genes (or additional markers) directly from a few known markers, or from brightfield images in the label-free case, some of the fluorescence channels become redundant. Thus, they are freed and can be used with different stains in order to study other cellular properties and functions. This was the case in the human data, where for example CD4 was not measured in the experiment but CD4 positive cells were identified by IFC-seq. Second, there are cases where avoiding certain fluorescent stains may be a goal in itself due to potential side effects of the staining process. The above advantages become especially pronounced in the label-free case, where analysis methods rely on cellular morphology (25,27), subcellular structures (61) or other label-free modalities (62). Moreover, label-free cell phenotyping has the potential to speed up and significantly lower the costs of routine diagnostics (63) Last but not least, it should be noted that the mouse myeloid progenitor dataset used to showcase the label-free mode of IFC-seq is a particularly challenging use-case, since it has been previously shown that most CMP cells are nearly indistinguishable from their offspring GMP and MEP populations based only on morphological information, with the exception of CMP cells that are close to being differentiated (29). Supplementary Figure S4 shows exemplary brightfield images of CMP, GMP and MEP cells, along with guided saliency maps (64) visualizing the pixels of each input image influencing the CNN’s predictions. The saliency maps were computed with keras-vis (https://raghakot.github.io/keras-vis) and suggest that all parts of the input image contribute equally to both CD34 and FcgR predictions and that the network mainly bases its predictions on regions near the cellular boundary and in some cases on regions deeper inside the cell.

To quantitatively validate the performance of the proposed method we need to be able to experimentally assess how accurately the predictions generated by IFC-seq reflect the ground truth gene expression at the single-cell level. To this extent, we would need an experimental procedure, which performs imaging and sequencing on the exact same cell in a high-throughput manner and results in a dataset in which both the IFC and SCT modalities are simultaneously measured for each cell. To the best of our knowledge, no such dataset exists at the moment but recent developments in next generation imaging and sorting techniques such as (30,31) suggest that this is only a matter of time. We expect that these new datasets where IFC and SCT modalities are simultaneously present will not only allow us to properly validate, but also improve the performance of the proposed methodology. Additionally, lower-throughput experimental methods capable of imaging and sequencing individual cells are currently available (65). Such methods are not practical in the label-free case where large datasets are required to train a CNN, but could be used to train and validate the performance of IFC-seq using the measured marker values. Having access to the expression values of key marker genes would be crucial for the validation of IFC-seq, especially in label free mode where the expression of key marker genes could be used as a control. Nonetheless, this requires some familiarity with the cellular populations at hand. Last, IFC-seq can also be extended to be useful in additional imaging modalities, other than IFC. That is, we expect IFC-seq will benefit from the advent of spatial transcriptomic methods (66,67) for spatially resolved transcriptional information in tissues. Using these next generation datasets it will be possible to predict gene expression directly from the imaging modality, without the need of an additional step of coupling different datasets using common surface markers.

## Supplementary Material

gkaa926_Supplemental_File
