# Supplementary material for: Predicting single-cell gene expression profiles of imaging flow cytometry data with machine learning
Source: Nucleic Acids Res. 2020 Oct 29;48(20):11335–46. doi: 10.1093/nar/gkaa926 (PMC7672460; doi:10.1093/nar/gkaa926)
Supplement: gkaa926_Supplemental_File [file gkaa926_supplemental_file.pdf]

Supplementary materials

In this section we provide supplementary material to the original manuscript.

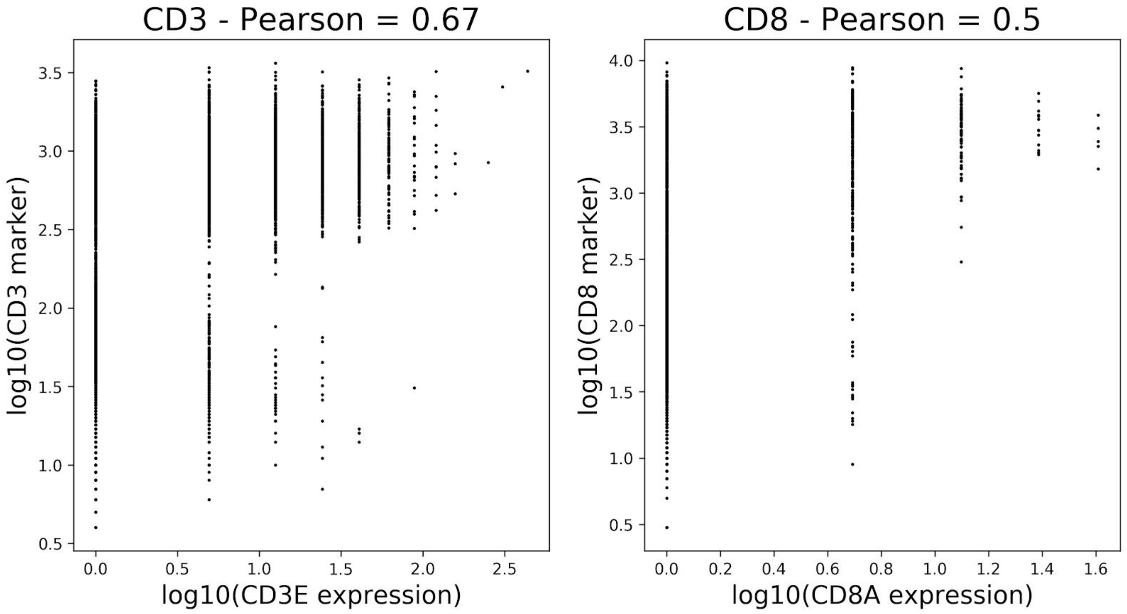

Figure S1. Scatter plots and correlation values for surface markers and their corresponding coding genes in the SCT dataset of human blood mononuclear cells. The y-axis corresponds to logarithmized values of surface markers while the x-axis corresponds to logarithmized counts of the marker's coding gene. In the case of CD3 (left), the Pearson correlation between the CD3 marker and its CD3E coding gene is 0.67, while for CD8 (right) the corresponding value of correlation to its CD8A coding gene is 0.5.

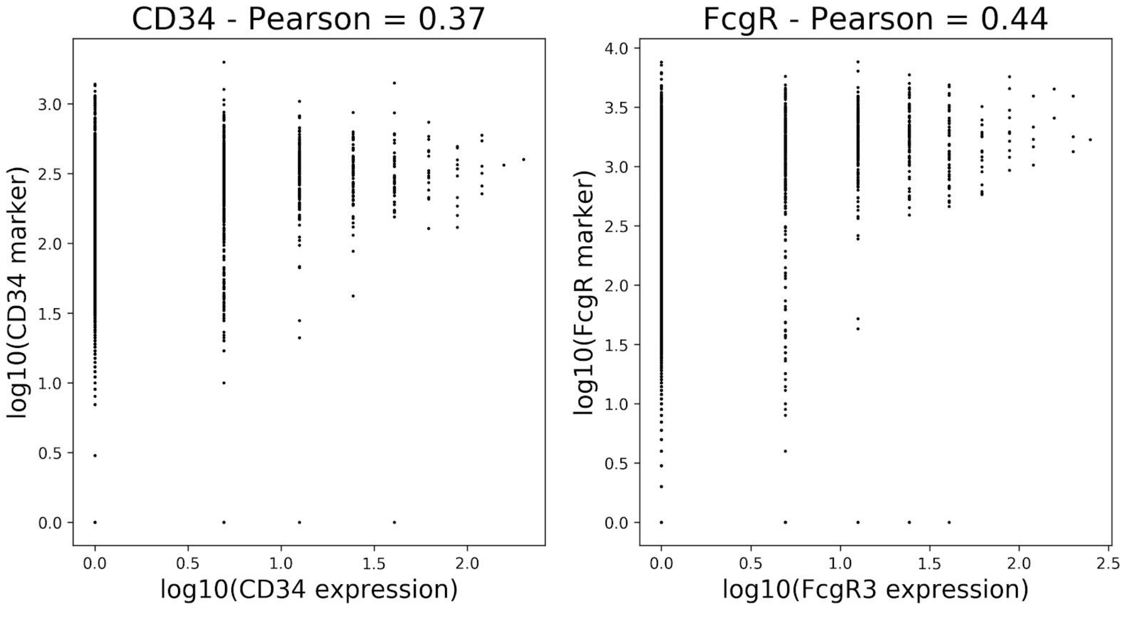

Figure S2. Scatter plots and correlation values for surface markers and their corresponding coding genes in the mouse SCT dataset of mouse myeloid progenitor cells. The y-axis corresponds to logarithmized values of surface markers while the x-axis corresponds to logarithmized counts of the marker's coding gene. In the case of CD34 (left), the Pearson correlation between the CD34 marker and its CD34 coding gene is 0.37, while for FcgR (right) the corresponding value of correlation between the FcgR marker and its FcgR3 coding gene is 0.44.

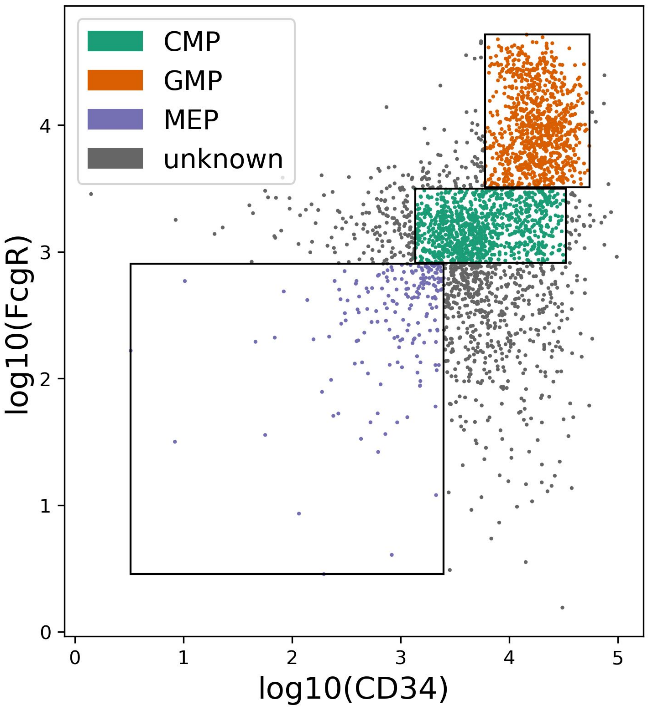

Figure S3. IFC data of mouse myeloid progenitor cells, plotted on top of the CD34 and FcgR markers, along with gates for the CMP, GMP and MEP populations of interest.

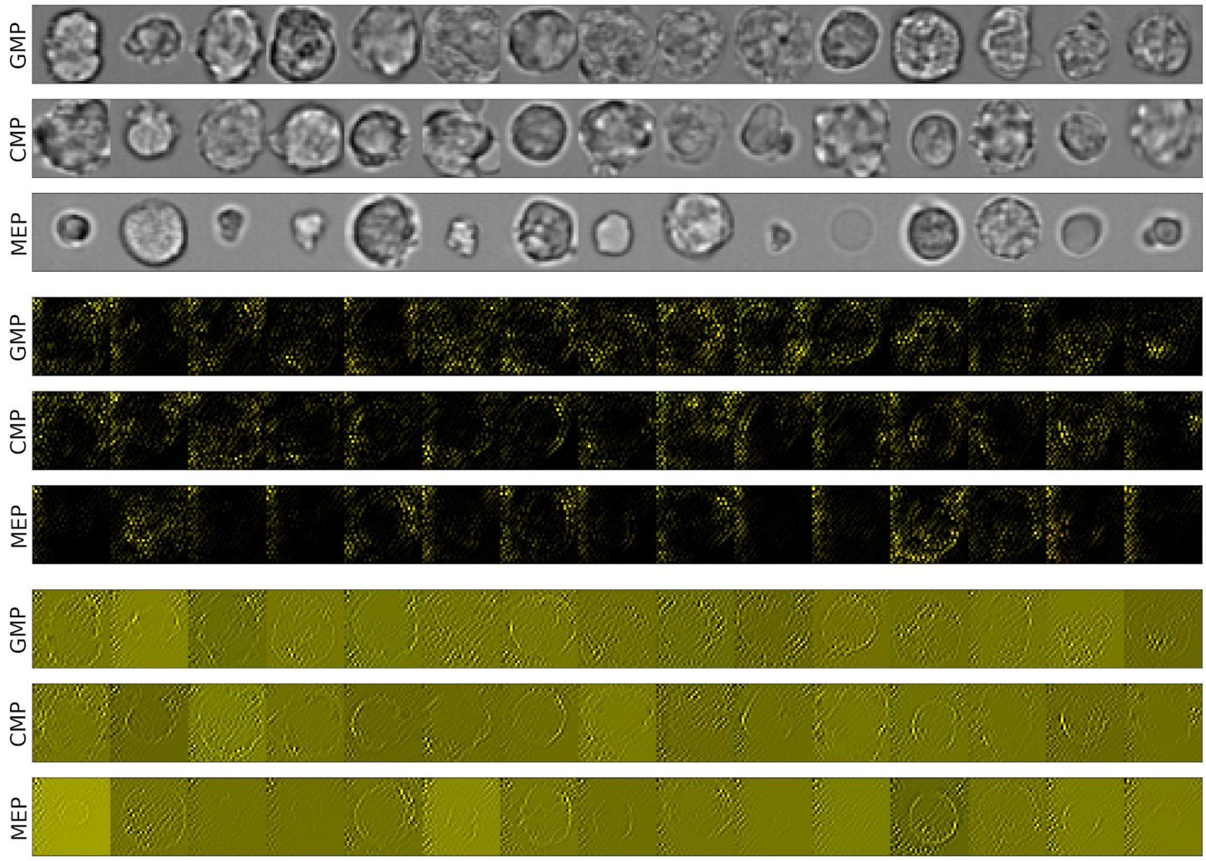

Figure S4. The cellular boundaries influence the network's predictions of surface markers. **Top:** Grayscale brightfield images of GMP, CMP and MEP cells of the mouse dataset. **Middle:** Saliency maps visualizing pixels with a positive association to the predicted marker. **Bottom:** Saliency maps visualizing pixels with a negative association to the predicted marker. In both cases brighter values correspond to a stronger association. In the case of saliency maps, the images are RGB where the saliency for CD34 corresponds to the red channel, the saliency for FcgR to the green channel and all pixels of the blue channel are set to zero. All saliency map images appear yellow, highlighting that pixels contribute equally to CD34 and FcgR predictions. As such, there are no areas that contribute to the network's decision on the value of a single individual marker. Last, saliency maps suggest that the network bases its decision mostly on features corresponding to regions near the cellular boundary and in some cases on pixels residing deeper inside the cell.
